# Medical genetics studies at BGRS conference series

**DOI:** 10.1186/s12881-019-0769-z

**Published:** 2019-04-09

**Authors:** Ancha V. Baranova, Mikhail Yu. Skoblov, Elena N. Voropaeva, Piramanayagam Shanmughavel, Yuriy L. Orlov

**Affiliations:** 1 School of Systems Biology, George Mason University, Fairfax, VA USA; 2 Research Centre for Medical Genetics, 115478 Moscow, Russia; 3 Research Institute of Internal and Preventive Medicine - Branch of the Institute of Cytology and Genetics SB RAS, 630090 Novosibirsk, Russia; 4 Bharathiar University, Coimbatore, Tamilnadu 641046 India; 5 Novosibirsk State University, 630090 Novosibirsk, Russia

This Special Issue of BMC Medical Genetics presents the papers materials discussed at the biomedical session of the multi-conference “Bioinformatics of Genome Regulation and Structure\Systems Biology” (BGRS\SB-2018) (http://conf.bionet.nsc.ru/bgrssb2018/en/). This international biannual conference takes place in Novosibirsk since 1998 gathering professionals in genomics, genetics, and biomedicine. To accompany this Special Issue, other Special Issues in the fields of genomics, bioinformatics, plant biology, evolutionary biology and systems biology are published as a part of following series: BMC Medical Genomics, BMC Bioinformatics, BMC Systems Biology, BMC Evolutionary Biology and BMC Plant Biology [1–4], as well as BMC Genomics and BMC Genetics issues. Medical genetics problems have been discussed in the articles from previous post-conference journal issues [5–8]. In year 2017, respective conference highlights were organized into the Special Issues with reports from Belyaev Readings-2017 memorial event (http://conf.bionet.nsc.ru/belyaev100/en) [9–12]. Note medical genetics topics from the conference series were presented earlier at BMC Medical Genomics [1, 12] and BMC Neuroscience [9] as well as BMC Genetics [8, 11] special issues.

The papers comprising this issue of BMC Medical Genetics were discussed at the BGRS\SB–affiliated symposium “Systems Biology and Biomedicine” (SBioMed-2018) (http://conf.bionet.nsc.ru/ishg2018/en/). A brief summary of these papers and related works is outlined below.

We start our Special Issue by the paper by Snezhkina and co-authors discussing novel potential causative genes in carotid paragangliomas [13]. Carotid paragangliomas are rare neuroendocrine tumors that arise from the paraganglion at the bifurcation of the carotid artery and are responsible for approximately 65% of all head and neck paragangliomas. Unlike other types of cancer, there is no test that determines benign from malignant tumors. Genetic basis behind the development of these tumors is not fully elucidated, and the molecular mechanisms underlying CPGL pathogenesis remain unclear [14]. Analysis of exome and transcriptome tumor samples allowed Snezhkina et al. to determine novel potential causative genes.

Yurchenko et al. [15] presented the exome-wide survey of the Siberian Caucasian population. This study has compared allele frequencies of Siberian Caucasian and European populations from 1000 Genomes Project and identified significant population differentiation as well as a higher proportion of the Finnish genetic component in the Siberian sample. Several of the allelic differences correspond to medically and pharmacogenetically important genes, and these variants will be studied in future on an expanded dataset with associated clinical data.

Maria Fedorova and colleagues [16] used colon adenocarcinoma TCGA RNA-Seq dataset to show that CpG island methylator phenotype, which is common in carcinomatous malignization of serrated adenomas, is associated with the shift in an energy metabolism and sufficient activation of immune-associated pathways. These findings were confirmed by qRT-PCR validation in additional cohort of patients. The ANDSystem tool for association studies in biomedicine was presented earlier at BMC Systems Biology special issues [17].

Diana Osmanova and her colleagues [18] provide us with insights into involvement of dopaminergic pathways in antipsychotic-induced hyperprolactinemia by uncovering statistically significant association between a certain polymorphic variant of gene MAOB, which encodes dopamine catabolizing enzyme and the development of hyperprolactinemia in response to any antipsychotic drug or their combination, and two other, risperidone/paliperidone–responsive variants in *SLC6A3,* which encodes dopamine transporter. Problem of depression studies were discussed by the same authors’ group at their recent article at Frontiers in Genetics [19], also being part of BGRS-2018 special issue.

Therapeutical strategies for anxiety-like behavior treatment continued by the works of Fedotova et al. [20]. Here Julia Fedotova discussed vitamin D3 treatment differentially affect anxiety-like behavior in the old ovariectomized female rats treated with low dose of 17β-estradiol [21]. After the menopause or surgical removal of ovaries, females are faced with estrogen deficiency, manifested in mood disturbances, such as anxiety and depression. Hormonal replacement therapy used to treat these symptoms has numerous side effects and not always efficient. Fedotova used rat model to evaluate the effects of repeated vitamin D3 administration on anxiety-related behavior in the middle-aged and old female rats with long-term estrogen deficiency. She determined that repeated systemic treatment with vitamin D3 decreased the symptoms on anxiety-like behavior in the old female rats after long-term ovariectomy. Extension of this work for human patients may provide a safe and efficient alternative therapy for many women.

Hence, we present our readers with a wide array of reports describing recent breakthrough in genomics-driven understanding of a molecular pathophysiology of a variety of human disease, covering a spectrum from Mendelian disorders to chronic multifactorial conditions and cancer. At BGRS-2018, the symposium “Systems Biology and Biomedicine” (SBioMed-2018) was also attended by young scientists who gathered in Novosibirsk for a School “Systems Biology and Bioinformatics” (SBB-2018) (http://conf.bionet.nsc.ru/bgrssb2018/en/school/). In previous years, the materials of SBB Schools were published in Special Issues of BMC as well [7, 22]. We invite our readers worldwide to attend our next event - Systems Biology and Bioinformatics Young Scientists School SBB-2019 which will be held in Novosibirsk, Russia 24-28 June 2019 (http://conf.bionet.nsc.ru/sbb2019/en/). Next BGRS\SB-2020 multiconference and biomedical symposium will be organized again in June 2020, in Novosibirsk, Russia.
